# Organotypic sinonasal airway culture systems are predictive of the mucociliary phenotype produced by bronchial airway epithelial cells

**DOI:** 10.1038/s41598-022-23667-y

**Published:** 2022-11-10

**Authors:** Juliette Delhove, Moayed Alawami, Matthew Macowan, Susan E. Lester, Phan T. Nguyen, Hubertus P. A. Jersmann, Paul N. Reynolds, Eugene Roscioli

**Affiliations:** 1 Adelaide Medical School, University of Adelaide, Adelaide, SA Australia; 2 Respiratory and Sleep Medicine, Women’s and Children’s Hospital, Adelaide, SA Australia; 3 Respiratory Department, Lyell McEwin Hospital, Adelaide, SA Australia; 4 Department of Immunology and Pathology, Monash University, Melbourne, VIC Australia; 5 Department of Rheumatology, The Queen Elizabeth Hospital, Adelaide, SA Australia; 6 Department of Thoracic Medicine, Royal Adelaide Hospital, Adelaide, SA Australia; 7 Adelaide Health and Medical Science, Building, Corner of North Terrace and George St, Adelaide, SA 5005 Australia

**Keywords:** Biological techniques, Cell biology, Physiology, Medical research

## Abstract

Differentiated air–liquid interface models are the current standard to assess the mucociliary phenotype using clinically-derived samples in a controlled environment. However, obtaining basal progenitor airway epithelial cells (AEC) from the lungs is invasive and resource-intensive. Hence, we applied a tissue engineering approach to generate organotypic sinonasal AEC (nAEC) epithelia to determine whether they are predictive of bronchial AEC (bAEC) models. Basal progenitor AEC were isolated from healthy participants using a cytological brushing method and differentiated into epithelia on transwells until the mucociliary phenotype was observed. Tissue architecture was assessed using H&E and alcian blue/Verhoeff–Van Gieson staining, immunofluorescence (for cilia via acetylated α-tubulin labelling) and scanning electron microscopy. Differentiation and the formation of tight-junctions were monitored over the culture period (day 1–32) by quantifying trans-epithelial electrical resistance. End point (day 32) tight junction protein expression was assessed using Western blot analysis of ZO-1, Occludin-1 and Claudin-1. Reverse transcription qPCR-array was used to assess immunomodulatory and autophagy-specific transcript profiles. All outcome measures were assessed using R-statistical software. Mucociliary architecture was comparable for nAEC and bAEC-derived cultures, e.g. cell density *P* = 0.55, epithelial height *P* = 0.88 and cilia abundance *P* = 0.41. Trans-epithelial electrical resistance measures were distinct from day 1–14, converged over days 16–32, and were statistically similar over the entire culture period (global *P* < 0.001). This agreed with end-point (day 32) measures of tight junction protein abundance which were non-significant for each analyte (*P* > 0.05). Transcript analysis for inflammatory markers demonstrated significant variation between nAEC and bAEC epithelial cultures, and favoured increased abundance in the nAEC model (e.g. *TGFβ* and *IL-1β*; *P* < 0.05). Conversely, the abundance of autophagy-related transcripts were comparable and the range of outcome measures for either model exhibited a considerably more confined uncertainty distribution than those observed for the inflammatory markers. Organotypic air–liquid interface models of nAEC are predictive of outcomes related to barrier function, mucociliary architecture and autophagy gene activity in corresponding bAEC models. However, inflammatory markers exhibited wide variation which may be explained by the sentinel immunological surveillance role of the sinonasal epithelium.

## Introduction

The possibility of making predictions of the lower airways using cells in the sinonasal compartment is extremely attractive as, (1) they require appreciably less intervention and time/cost resource to obtain which relaxes concerns for the participant and clinicians/physicians^1,2^, (2) this, in turn alleviates the issues surrounding recruiting control participants and widens disease-related inclusion criteria, and (3) in technical terms, sinonasal sampling is more reproducible and, in our experience, returns a higher yield of viable cells. Given the shared structure and function of the upper and lower epithelium lining conducting airways, it is reasonable to presume nAEC share the same omics-profiles and encounter similar exogenous (e.g. air, microbial, occupational) and endogenous (e.g. nutritional, immune, pharmaceutical) exposures as their lower airway analogues. This underlies the united airway hypothesis which considers the entire airway as a continuum due to the coincidence of sinonasal disease with conditions of the lower airways, and observations that treatment of one site can ameliorate symptoms in the other^3,4^. For example, observations in asthmatics show that up to 80% also suffer from chronic rhinosinusitis^5^, and treating both sites improves clinical outcomes and can reduce exacerbations vs targeting them individually^6^. Of course, this can be explained by (for example) abatement of pro-inflammatory and damage associated molecular factors that originate from the primary site of disease vs phenomena that are transmitted through the airway epithelium. Further, the sinonasal and lower airways have very different gross anatomical structure, distinctions in temperature and humidity^7^, and their relative location means nAEC are the first to encounter environmental challenges^1^.

Nevertheless, it would be a major advantage if samples from the sinonasal cavity proved to be diagnostic (and/or prognostic) of lower airway disease. A recent study applied RNA-Seq technology to assess paediatric samples in the context of the unified airway hypothesis, and identified 91% homology of gene transcript profiles between matched bronchial and nasal cell isolates, with gene activity outcomes aligning with wheeze symptom scores^8^. While a similar investigation in adults would likely resolve further differences due to increased incidence of disease and years of environmental/occupational exposures, this study provides evidence that nAEC may be well suited to proxy the lower airways for paediatric patients. Indeed, a study by Imkamp et al. in the context of COPD, identified appreciable overlap in gene expression and quantitative trait loci associated with cigarette-smoke exposure^9^. However, they also noted frequent gene-dependent observations (as did the Kicic et al. 2020 study), that highlighted significant distinctions between the nasal and bronchial compartments. Gene-specific differences have been reported in at least three further transcriptome-based studies; one investigating whether bAEC expression signatures could serve as a predictor of cigarette smoke-induced damage to the lower airways^10^, another for the early diagnosis of lung cancer^11^, and in the context of cystic fibrosis, Ogilvie et al. concluded that nAEC are not reliable surrogates for the lower airways^12^. Discordance among investigations that assess clinical isolates (vs cultures) gives reason to the absence of clinical (or diagnostic) applications that may be used to predict lower airway disease with sinonasal biopsies. Bourdin et al. provides an excellent discussion of the considerations that disconnect the nasal and bronchial epithelium in the context of the unified airway disease hypothesis^5^. Perhaps the most significant being that the nasal airway epithelium encounters and mitigates significantly more environmental exposures than that of the lower airways. Indeed, the immunological consequences leading to COVID align with a high load of SARS-CoV-2 within the sinus epithelium vs the lower airways^13^. Altogether, these reports point towards differences that give rise to heighten resilience and immunological surveillance for nAEC vs their lower airway counterparts.

Another consideration is whether nAEC are valid surrogates for bAEC in laboratory models, and thereby provide a generally applicable (and affordable vs commercial sources), ex vivo model of the airways with greater predictive qualities than current options. nAEC are objectively better than bAEC lines which (for example) require artificial genetic aberrations (e.g. unlimited proliferation in non-physiological media), and they retain few characteristics synonymous with normal physiological activity. For example, we have shown that primary nAEC models respond more effectively to microbial exposures and produce distinct secretory profiles compared to bAEC lines purported to be “close to normal”^14^. Despite this, it is difficult to reconcile the diverse outcomes from the paucity of investigations that directly compare nAEC and bAEC models. Mihayola et al. compared undifferentiated models (albeit using commercially sourced primary cells), and convincingly showed differences in *RIG1* activity, coupled with distinct interferon and survival responses, that confer heightened antiviral activity in nAEC cultures^15^. Divergent outcomes have also been reported for the production of influential inflammatory factors (e.g. IL-6 and TLR-4) by Comer et al., who cautioned against applying nAEC cultures in the context of COPD^16^. In contrast, McDougall et al. found significant correlation for inflammatory profiles between submerged nasal and bronchial cultures (stimulated with e.g. IL-1β and TNFα), despite using a heterogenous cohort of non/smokers with a range of respiratory conditions^17^. However, even fewer studies apply a tissue engineering approach to assess organotypic models, which is essential to emulate (for example) omics profiles and mucociliary function observed in vivo^2,18,19^. To our knowledge, this has been performed rigorously on one occasion using cystic fibrosis paediatric samples differentiated at ALI^20^. While they demonstrate nAEC and bAEC share comparable electrophysiological properties, the authors cautioned that it is less likely that these results inform nAEC as surrogates in conditions that bias lower airway disease, and that their use of conditionally reprogrammed cells may have diminished cell-specific differences in gene activity.

A likely scenario is that nAEC and bAEC share a great majority of genetic and biochemical processes, but which become disparate due to modifications brought about by age, exposures and disease. Hence, here we apply a first principles approach and assess organotypic ex vivo models of the sinonasal and bronchial airway epithelium derived from healthy participants. Our findings show that the models are indistinguishable in terms of morphology and barrier function, and this may also be the case in vivo. Conversely, transcript abundance of inflammatory markers demonstrated appreciable variation between the cultures, particularly in contrast to genes that regulate the evolutionarily conserved process of autophagy.

## Results

### Differentiated nasal and bronchial AEC cultures exhibit shared tissue architecture

**Figure 1:**
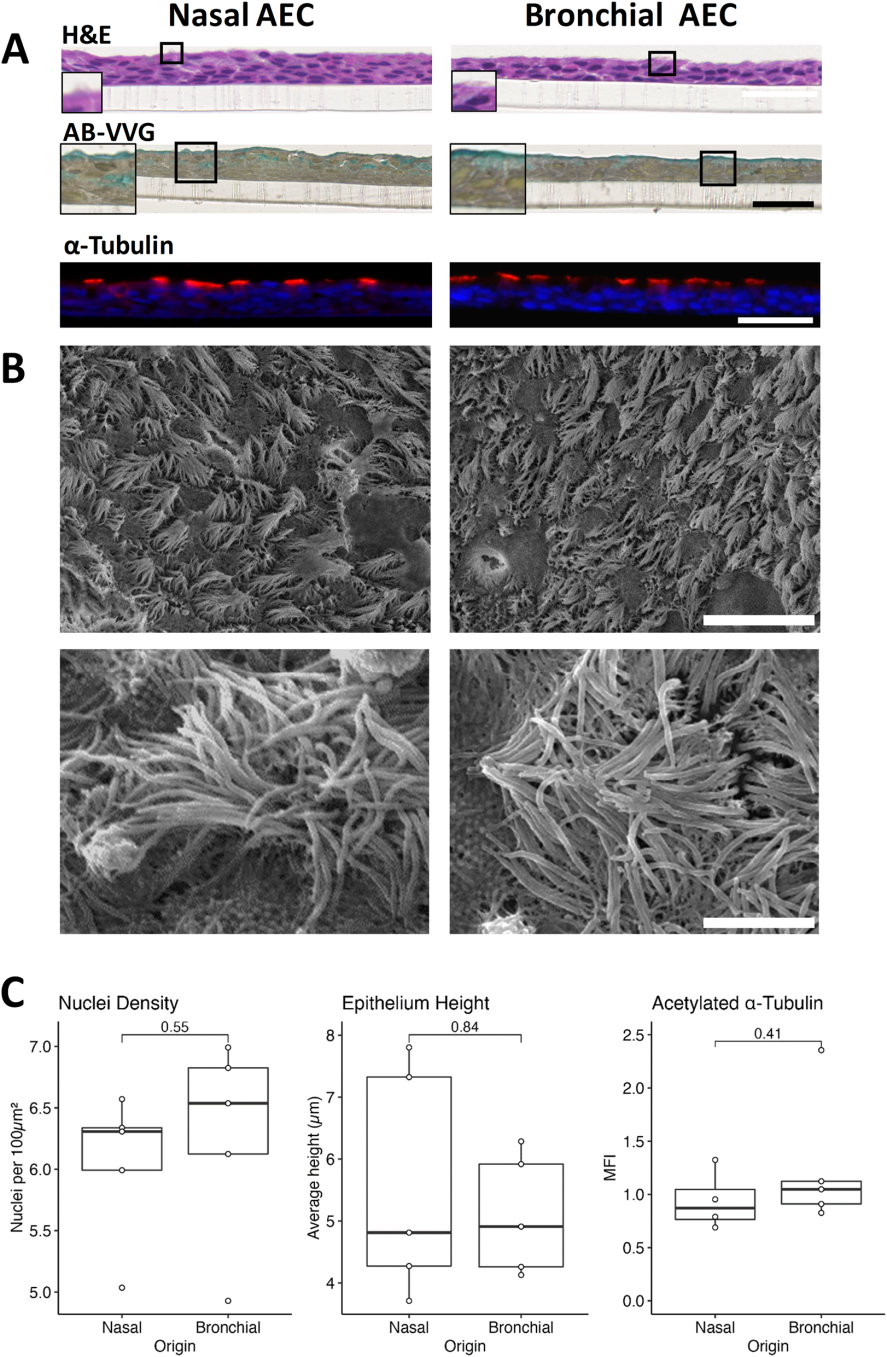
Nasal and bronchial progenitor cells produce similar tissue architecture when differentiated at an air–liquid interface. Comparative microscopic analysis of air–liquid interface (ALI) tissue engineering models for nasal and bronchial airway epithelial progenitor stem cells. Both were grown for 4–6 days submerged, followed by a further thirty-two days at an ALI. (**A**) Immunohistochemical assessment shows that nasal and bronchial ALI cultures share gross morphology and histological features. Hematoxylin and eosin (H&E) revealed similar three dimensional tissue architecture, and alcian blue/Verhoeff–Van Gieson staining resolved mucin (blue) in mucus producing cells and in a continuous apical airway surface liquid layer. Immunofluorescence analysis of cilia via acetylated α-tubulin (red channel) shows similar apical localisation and an interrupted lateral distribution consistent with the presence of interspersed goblet and brush cells. The blue channel shows cell nuclei via 4′,6-diamidino-2-phenylindole staining. Scale bar is 50 microns. The transparent structure under the cell layer is the transwell membrane. Images are representative of n = 5 cultures. Scale bar is 50 microns. (**B**) Representative scanning electron microscopy images (lower four panels) show similar density, clustering and distribution of cilia for either culture (upper images; 2000 × magnification; scale bar is 50 microns). Closer examination (lower panels, 10,000 × magnification; scale bar is 5 microns) shows the cilia are of similar structure and length (6–8 microns) for both nAEC and bAEC cultures. Also in both cultures are squat microvilli-like projections indicative of brush cell (lower images). Two further participant SEM micrographs are provided in Supplementary Fig. S1 online. (**C**) Quantification of H&E micrographs resolved no significant difference in cell architecture for measures of cell density and epithelial height (right and middle graphs), between nasal and bronchial cultures. Similarly, outcome measures for mean fluorescence intensity (MFI, right graph) of labelled acetylated α-tubulin, as a specific marker of cilia, were approximately the same for both AEC culture models. Confidence intervals are SEM; n = 5 per culture group.

We coupled the ALI culture model with microscopic analysis techniques and assessed the comparative structure of differentiated nAEC vs bAEC epithelia (Fig. 1). Histological examination showed that either progenitor stem cell population, when cultured on transwell membranes, effectively differentiate to form a three dimensional tissue architecture (100% success rate), which enabled the production of the mucociliary phenotype (Fig. 1A). For all samples (and visual fields), hematoxylin and eosin (H&E) staining resolved ciliated cells, and alcian blue/Verhoeff–Van Gieson staining resolved mucus-producing goblet cells which formed a continuous airway surface fluid layer (blue staining). Observations made during routine light microscopy showed that nAEC produced detectable amounts of airway surface fluid earlier (approximately day 8–10, vs 12–14 days for bAEC), whereas ciliogenesis (and cilia beating) was first seen for the bAEC cultures (day 14–16, vs 16–18 for the nAEC). Quantification of the H&E stained sections returned no significant difference for measures related to cell density (nuclei per µm^2^; nasal = 6.048/μm^2^ vs. bronchial = 6.281/μm^2^, 95% CIs [5.520, 6.58], [5.59, 7.00], *P* = 0.55, n = 5) and the width of the epithelial layer (µm basal to apical cell margin; nasal = 5.585 μm vs. bronchial = 5.11 μm, 95% CIs [3.96, 7.21], [4.25, 5.95]; *P* = 0.84; n = 5; Fig. 1C). As both nAEC and bAEC-derived epithelia exhibited similar cell density, we measured a fluorescence signal directed to acetylated α-tubulin to compare their respective ciliogenic potential. There was similarity between the frequency of cilia quantified for either culture (Fig. 1A,C; MFI of acetylated α-tubulin; nasal = 0.94 vs. bronchial = 1.25, 95% CIs [0.69, 1.18], [0.70, 1.80], *P* = 0.41, n = 5), which was consistent with the magnitude of difference observed for cell density. These results also indicate a proportionally similar frequency of ciliated AEC, and cilia per AEC, shared by these cultures. Observations from high sensitivity microscopy (SEM) confirmed that the distribution of cilia was approximately equal for either AEC culture, and that cilia morphology (e.g. consistently > 6–8 microns in lengths) and clustering were indistinguishable between the two models (Fig. 1B).

### Differences in barrier activity converge and approximate during differentiation

**Figure 2:**
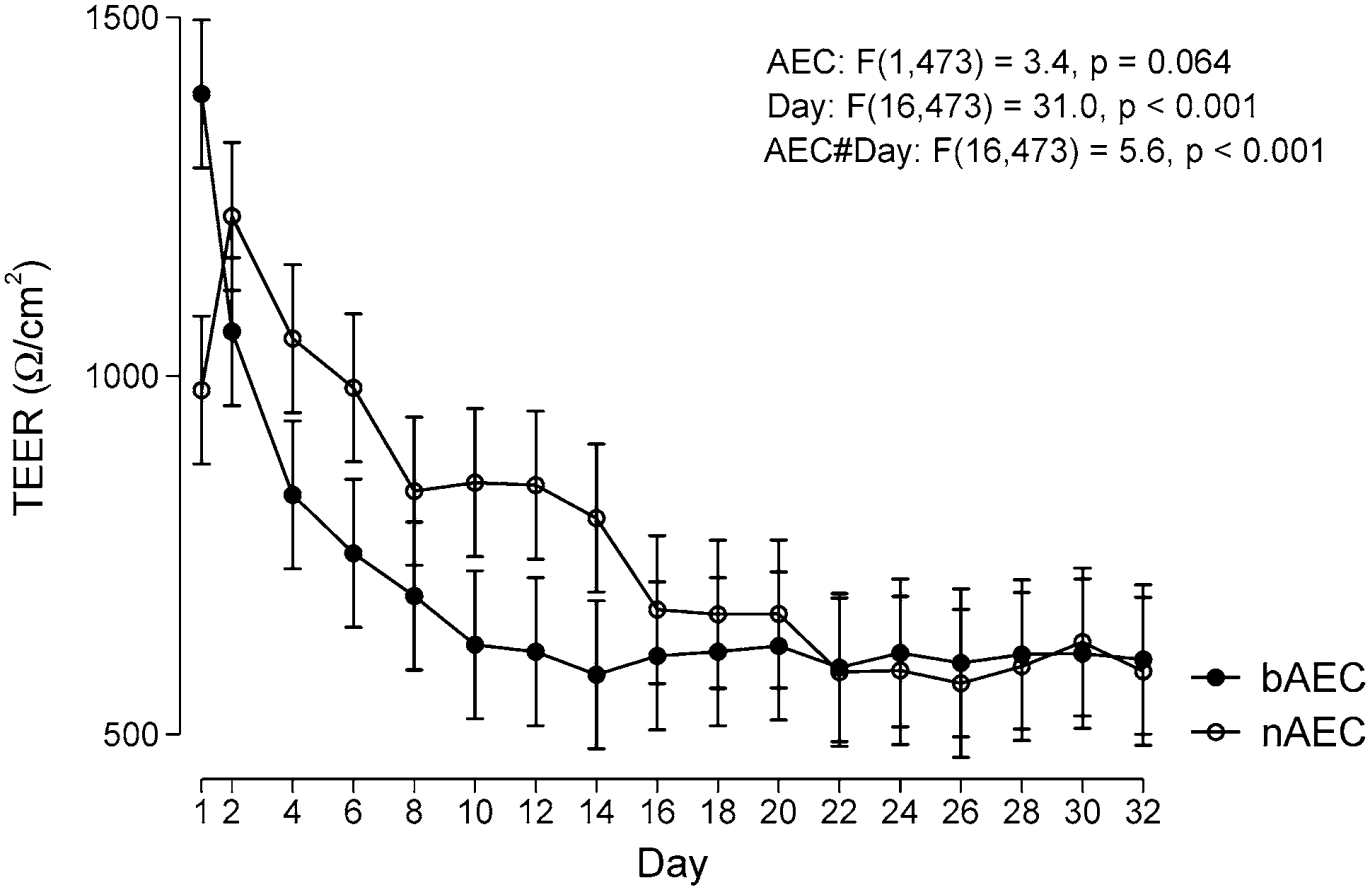
Barrier activity imparted by nAEC and bAEC epithelia aligns sixteen days after air-lift. Trans-epithelial electrical resistance (TEER) was assessed/compared in cultures of nAEC and bAEC as they differentiated into epithelia in the transwell system. Day 1 represents 24 h after the growth media was removed from the apical reservoir (“air-lift”). TEER was measured three times at each time point, every 48 h, for any one culture (n = 5 per participants per culture group) using each of the three ports in the transwell frame. TEER measures are expressed as resistance over the total membrane/cell coverage area (Ω cm^2^). Results are presented as marginal means predicted from a multilevel linear mixed model with 95% confidence intervals. The time course is different between bAEC and nAEC (P < 0.001), individually significantly different between days 1–14 (*P* < 0.05, Wald tests), and converging thereafter.

Critical to the airway epithelium are distinct cell types which interact via tight junction (TJ) complexes to coordinated emergent barrier properties and the mucociliary escalator. Hence, we assessed the formation of TJ by quantifying ion conductance of the paracellular pathway as basal progenitor nAEC and bAEC differentiated to produce apical ciliated and goblet cells. Interestingly both cultures return high trans-epithelial electrical resistance (TEER) values (days 1–14) that converge and stabilise to approximately 625–650 Ω cm^2^ after the day-16 observation interval (*P* > 0.05 from day 16–32 when comparing magnitudes for nAEC and bAEC-derived cultures). We were unable to determine whether this phenomena represents an artefact of cellular turnover that restricts ionic passage until differentiation starts to occur, or whether this reflects a physiological response indicative of a distinctions in a wound healing-like process (Fig. 2). With the exception of day 1 (24 h post-airlift), TEER measures before the 16 day interval show that the nAEC cultures are significantly more effective in impeding the flow of ions through the paracellular pathway. This may reflect heightened goblet cell activity for these cultures, as routine light microscopy resolved more secretion products for the nAEC cultures, and which were noticeably more difficult to clear using PBS (e.g. cilia beating was more difficult to resolve unless the PBS wash step was performed). Why the day 1 observation is contrary to this pattern is unclear. Given that the convention is to challenge (and/or apply end-point analyses) to ALI cultures 28–32 after days after air-lift, means that either may be used to predict phenomena related to TJ activity and barrier function.

### The abundance of essential tight-junction complex proteins are comparable between nAEC and bAEC cultures

**Figure 3:**
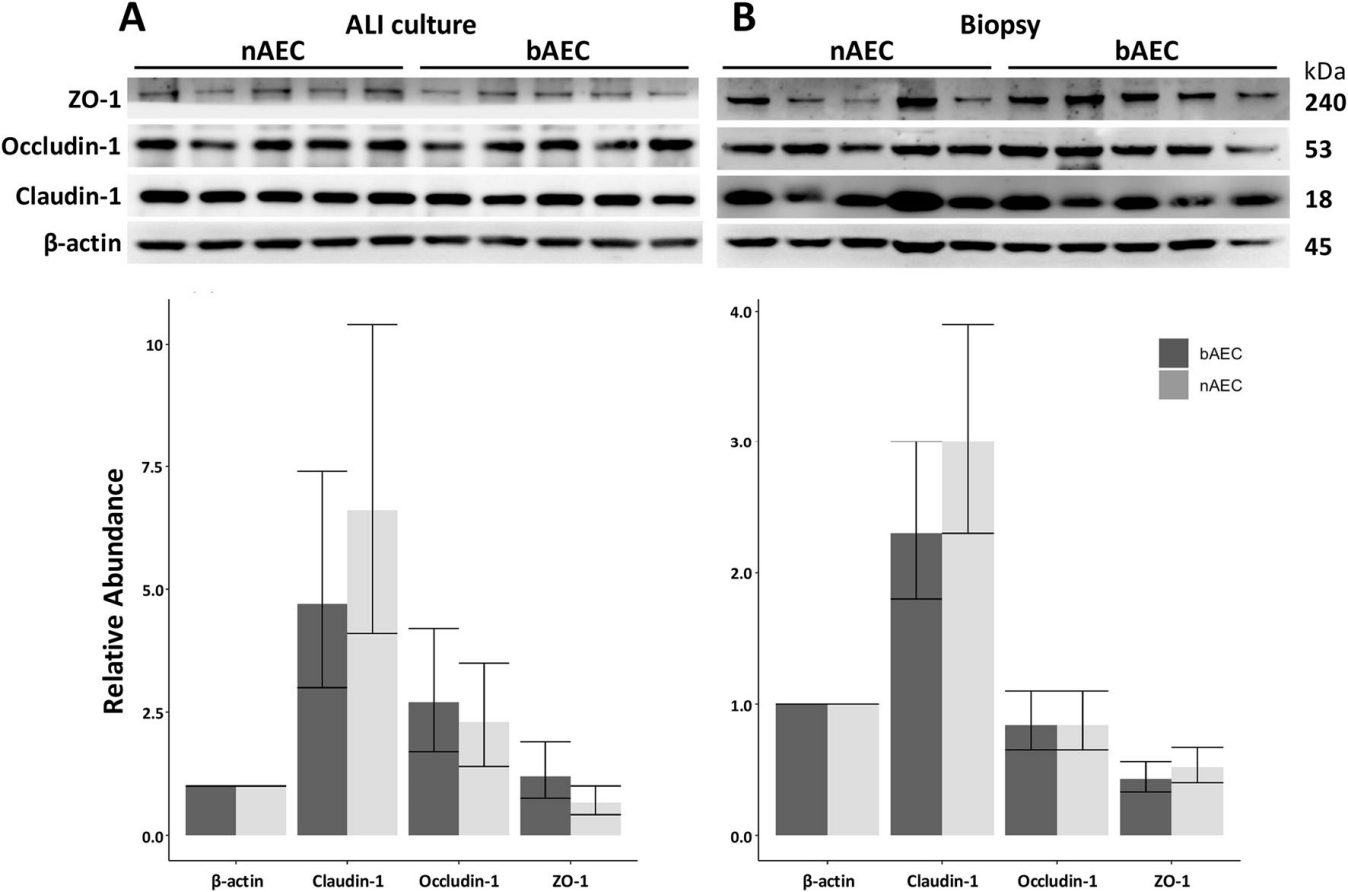
Tight junction proteins are similarly expressed in nAEC and bAEC cells for ALI and biopsy epithelia. (**A**) Ex vivo cultures of differentiated nAEC and bAEC express similar proportions of the TJ proteins Zonula occludens-1 (ZO-1), Occludin-1 and Claudin-1. (**B**) A portion of the biopsy sample used to generate the ex vivo models also produced no significant difference for the TJ factors. Blots were sectioned to probe the area corresponding to the molecular weight of the target protein. With the exception of β-actin for the ALI culture Western blot in (**A**), outcomes were derived from a single transfer membrane for the respective experiments (i.e. ALI vs biopsy) to minimise inconsistencies produced by inter-blot variation. Original blots are in Supplementary Fig. S2 online. Uncertainty intervals are 95% CI; n = 5 per culture group. Values were normalised to the β-actin loading control.

The relative abundance of individual components of the TJ apparatus was assessed between cultures at the end of the observation interval (day 32). There was no significant distinction identified for either TJ protein examined for the nasal and bronchial-derived epithelial cultures (Fig. 3A). However, the prolonged period (out of the in vivo situation) required to differentiate progenitor AEC into epithelia may attenuate the differences in nasal vs bronchial-derived stem cells beyond the sensitivity afforded by Western blot analysis. To mitigate this, TJ proteins were also assessed using AEC biopsy samples isolated directly from the bronchoscopy brush. A similar result (as the ex vivo model) was resolve for TJ protein abundance from the nasal sinus and bronchus sourced directly from the airways, and a similar pattern of expression was seen for each TJ factor (Fig. 3B). This result suggests that the conditions used to generate differentiated organotypic epithelial cultures does not distort the relative abundance of TJ proteins observed in participant-derived airway samples.

### Inflammatory-gene activity shows greater variation compared to the genes that govern autophagy

**Figure 4:**
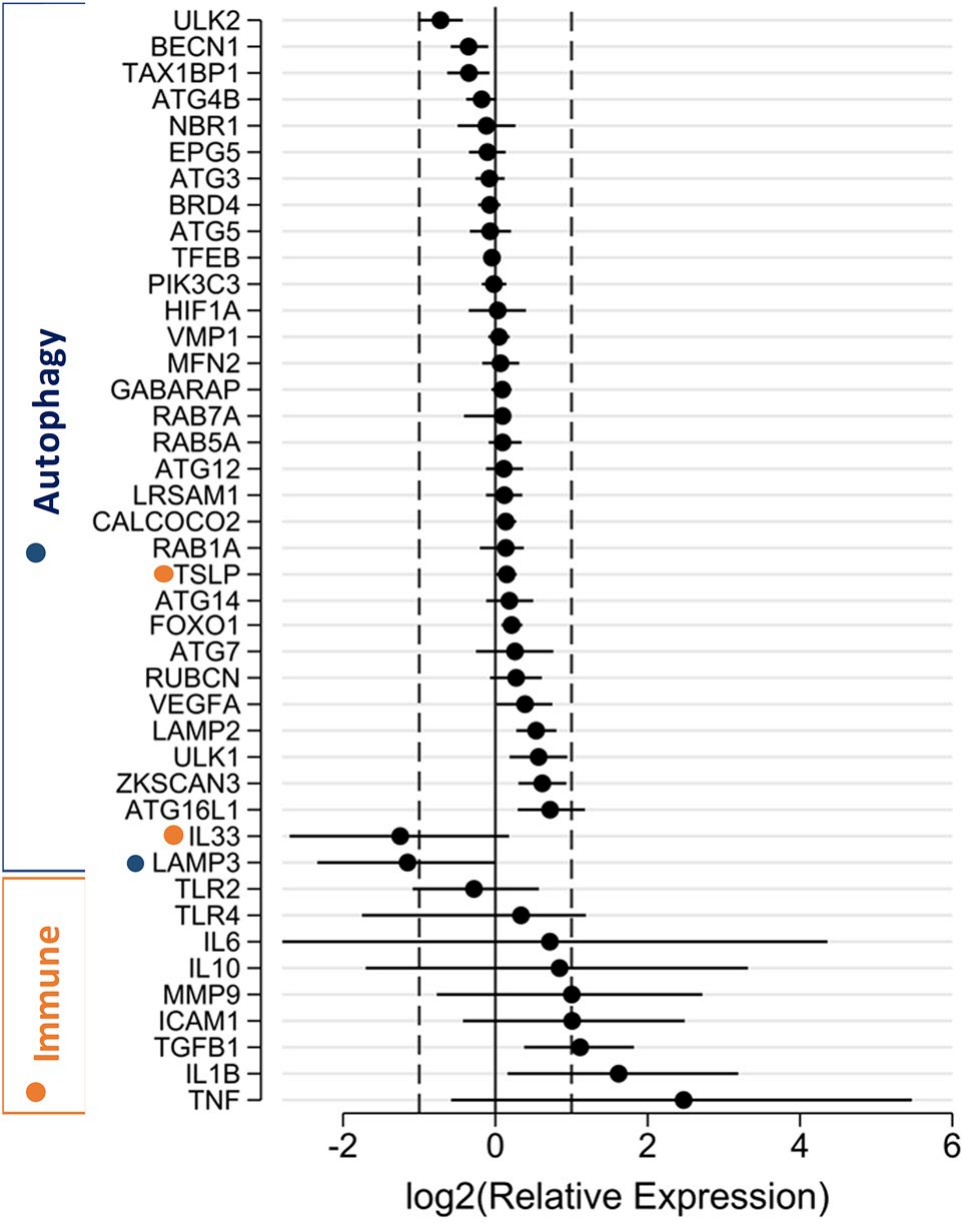
Differentiated nAEC and bAEC exhibit high variation in inflammatory gene activity *vs* those governing autophagy. Differentiated ex vivo models of the nasal and bronchial airway epithelium were assessed for the relative abundance of inflammatory and autophagy transcripts (32 days culture). Magnitudes are expressed as nAEC *vs* bAEC (less/more transcript abundance in nAEC-derived cultures to the left/right side of zero, respectively), and ordered to highlight transcript groups related to gene-dependent variation. Dashed vertical lines at log2(Relative Expression) at − 1.5 correspond to a fold-change of 0.35, and + 1.5 correspond to a fold-change of 2.8. Results (n = 5 participants per culture type) are normalised to three reference genes and are significant to at least *P* < 0.05 when 95% CI do not cross zero.

The products of inflammatory-gene activity with major significance to the nasal and bronchial airway epithelium were compared using a rational transcript analysis experimental design. Given inflammatory genes are highly inducible and subject to variation, the evolutionary conserved process of autophagy (present in all kingdoms except bacteria) was assessed simultaneously. Shown in Fig. 4, the relative abundance of inflammation-related and autophagy transcripts were similar for either cell/culture type (indicated with dashed lines in Fig. 4), with the exception of *TGFβ* and *IL-1β*, which exhibited a modest increase in the nAEC model (*P* < 0.05). In contrast, with the exception of *LAMP-3*, the gene activity for the regulators of autophagy was tightly controlled as indicated by similar effect sizes among the study cohort (Fig. 4), and confined effect-size distribution (i.e. broader uncertainty intervals) observed for the transcripts related to inflammation.

## Methods

### Primary human airway epithelial cell air–liquid interface culture model

Ethics approval to perform airway brushings was from the Central Adelaide Local Health Network Human Research Ethics Committee. Methods were conducted in accordance with the Declaration of Helsinki and with the understanding and the written consent of each participant. Airway brushing was performed by the physicians at the Royal Adelaide Hospital’s Department of Thoracic Medicine. Nasal and bronchial samples were obtained from consenting participants with no history of chronic respiratory disease and were never smokers (n = 10; FEV1/FVC median 93% ± 12 [SD], three female, median age 42 ± 16 years [SD]; Table 1). Human primary nasal epithelial cells were sampled from the inferior turbinate mucosa via nasal brushings and bronchial epithelial cells were obtained from the right main bronchi using bronchoscopy and catheter brush. Bronchial and nasal AEC isolates were subject to the same culture condition, as previously described^21^. Briefly, AEC were rinsed from the sampling brush into complete RPMI media, pelleted (300 g, 7 min, 10 °C), and resuspended in Bronchial Epithelial Growth Media (Lonza, Mt. Waverley, VIC, Australia). Cell suspensions were propagated in collagen coated T25 flasks (Sigma-Aldrich, Castle Hill, NSW, Australia) to expand basal progenitor AEC. Monolayers achieved 90% growth density after 8–12 days and were transferred to collagen coated (StemCell Technologies, Sunrise Beach, QLD, Australia) transwells (0.4 µm pores, 6.5 mm diameter; Sigma-Aldrich) and grown in ALI Growth Media (Lonza), at a seeding density of 9 × 10^4^ cells per well. Once cells achieved 100% density (4–6 days), media was removed from the apical and basal reservoirs, and bronchial ALI Differentiation Media (Lonza) was added to the basal reservoir. Media was changed every second day and the apical surface was cleared of excess mucus every 4 days with three 30 s washes using PBS. Cultures were included in ex vivo models when mucociliary differentiation was observed, and transepithelial electrical resistance measures (EVOM2; World Precision Instruments, FL. USA) exceeded 500 Ω cm^2^ on the final culture day (32 days).

**Table 1:** Participant demographics.

Tissue source	Gender	Age	FEV1/FVC (%)
Nasal	M	43	85
	M	65	77
	M	53	80
	M	25	101
	F	41	93
Bronchial	M	53	94
	M	27	99
	M	22	104
	F	34	93
	F	68	71

### Immunohistochemistry

Cells cultured for 32 days at an ALI were prepared for staining with the University of Adelaide Histology group. Briefly, cells were fixed in situ in the transwell system with 10% neutral buffered formalin (1 h), after which the membrane/cells were cut from the transwell frame for dehydration (ethanol gradient) and embedded in paraffin wax. Longitudinal sections of the transwell membrane were cut into 0.4 µm sections and mounted onto slides. After xylene dewaxing and ethanol/water rehydration, H&E and Alcian Blue Verhoeff Van Gieson staining was performed by the SA Pathology Immunohistochemistry Department (Royal Adelaide Hospital). The cells were imaged using NDP.view2 software (version U12388-01) and the NanoZoomer automated slide scanner platform (both Hamamatsu Photonics, Higashi-ku, Hamamatsu City, Shizuoka Pref., Japan). NanoZoomer light microscopy images of H&E-stained ALI sections were imported into ImageJ (version 1.52r) and their sizes calibrated (pixel to µm). ImageJ colour thresholding and particle analysis methods were used to count the number of nuclei per unit area. Nuclei density was then reported as nuclei per 100 µm^2^. Average height for each ALI culture section was determined by first taking the area of the section, and then dividing this by the width of each section to give the average value for the height of the entire section in µm.

### Immunofluorescence

Transwell sections (prepared as above) were brought to water using the Histoclear II (National Diagnostics, Atlanta, GA, USA) and a reducing ethanol gradient. Heat mediated antigen retrieval was performed in sodium citrate buffer (10 mM sodium citrate, 0.05% Tween 20; pH 6.0). Samples were permeabilised in TBS-Triton X-100 (0.3% for 15 min) and blocking for 1 h at room temperature in Dako serum-free protein block (SFB; Agilent, Santa Clara, CA, USA) containing 5% normal goat serum (VectorLabs, Burlingame, CA, USA) and 0.1% Tween 20. To detect cilia, sections were incubated with rabbit anti-human acetylated α-tubulin (1:500; Cell Signaling Technologies, Danvers, MA, USA) overnight at 4 °C followed by secondary antibody (goat anti-rabbit Alexafluor 568; 1:700) (ThermoFisher Scientific, Waltham, MA, USA) for 1 h at RT. Sections were mounted with Prolong Diamond antifade (ThermoFisher Scientic, Waltham, MA, USA) containing 4′,6-diamidino-2-phenylindole counterstain to resolve nuclei. Images were acquired using a Nikon Eclipse Ts2 microscope with NIS-Elements imaging software (version 5.20.00; both Nikon, Tokyo, Japan). Five randomly selected microscopy fields were used to determine mean fluorescence intensity of acetylated α-tubulin for the nasal- and bronchial-derived epithelial cultures.

### Scanning electron microscopy

High-resolution topographical epithelial morphology was assessed in situ with a previously described method using scanning electron microscopy (SEM)^22^. Briefly, fixative agents were applied directly to the cells on transwell (3 h) at the end of the culture period. The transwell/cells were then excised from the transwell frame, dehydrated and coated with platinum to for ultra-high resolution imaging to discern individual cilia. Coated samples (n = 2 each from nAEC and bAEC-derived cultures) were mounted on to SEM pegs and imaged using the FEI Quanta 200 scanning electron microscope (FEI Australia Pty Ltd, Canberra, ACT, Australia) housed in the University of Adelaide Microscopy Suite.

### Measurement of trans-epithelial electrical resistance

Electrical impedance (to restrict ionic conductance) imparted by epithelial cultures was measured to quantify barrier function (TJ activity), as previously described^19,23^. One difference here was the models were assessed during the entire differentiation process from day 1 (24 h after “air-lift”) to day 32. Briefly, transwell ALI cultures were allowed to acclimatise for 30 min on a 37 °C heating platform (Lecia Biosystems, Mt. Waverley, VIC, Australia) before reading electrical impedance using the EVOM2 Ohm meter (World Precision Instruments, Sarasota, FL, USA). Raw ohm values (n = 3 measures per well) were converted to magnitudes of TEER by subtracting the resistance imparted by an empty transwell and dividing by the surface area of the membrane support (0.33 cm^2^; Ω.cm^2^). Five transwells were prepared and assessed per participant per culture type.

### Western blot

As previously described^24^, protein was isolated from differentiated AEC cultures in situ from the transwell membrane surface using M-Per mammalian cell protein lysis reagent and Halt® protease and phosphatase inhibitor cocktail (both Thermo Scientific, Victoria, Australia). Protein samples were quantified using the BCA protein assay method (Bio-Rad, Victoria, Australia), and 10 μg electrophoresed using Novex® 4–12% gradient Bis–Tris denaturing gels (Life Technologies, Victoria, Australia) and electroblotted to Trans-Blot® Turbo nitrocellulose membranes (Bio-Rad). Membranes were blocked in 5% diploma skim milk and probed overnight at 4 °C with primary antibodies directed to Claudin-1 (37–4900), Occludin-1 (71–1500), ZO-1 (33–9100) (all Thermo Fisher Scientific, Waltham, MA, USA), or β-actin (A1978; Sigma-Aldrich Co., St Louis, MO, USA). Secondary antibody incubation was 1 h at RT with horse radish peroxidase-labelled secondary antibody (R&D Systems, MN, USA). Chemiluminescent imaging was performed using the LAS-3000 platform and histogram densitometry was performed using Multi Gauge software (V3.1 Fujifilm, Tokyo, Japan).

### Reverse transcription qPCR array analysis

Quantitative reverse transcription real-time PCR was used to identify differences in transcript abundance in differentiated nasal and bronchial cultures. RNA was extracted and genomic DNA eliminated from cell in situ while on the transwell membranes using the RNeasy Plus column system following the manufacturers protocol (QIAGEN, Chadstone, VIC Australia). Purified RNA was quantified using the NanoDrop One spectrophotometer (Thermo Scientific). RNA (1 µg) was subject to a second gDNA purification and reverse transcribed into cDNA with SuperScript IV VILO ezDNase (Thermo Scientific) using an Eppendorf Mastercycler (Eppendorf, Hamburg, Germany). cDNA samples (and reaction mix) were entered into a custom TaqMan low density array (Thermo Fisher Scientific Australia, Scoresby, VIC, Australia). qPCR was performed with 500 ng cDNA using TaqMan primer/probes technology in conjunction with TaqMan Fast Advanced Master Mix chemistry, in the microfluidics system (all Thermo Scientific). Thermocycling was performed using the ViiA7 7300 Real Time polymerase chain reaction platform (Applied Biosystems, Carlsbad, California, USA). Three internal reference genes were included to normalise outcomes across the experimental groups: *RNA18S5* (Hs99999901_s1), *HMBS* (Hs00609297_m1) and *TBP* (Hs00427620_m1). The complete set of primer/probes are shown in Supplementary Table S1 online.

### Statistical methods

Immunofluorescence and IHC outcome results were analysed with R (v 4.0.5) using the ggpubr R package (v 0.4.0) using non-paired Wilcoxon tests. TEER results and Western blot densitometry results were reanalysed using multi-level generalised linear mixed models (GLMM) in Stata v 16 (StataCorp LLC, TX, USA). TEER results were analysed as a linear glmm with measurement times (days) as repeated measures within participants. Results were expressed as predicted marginal means with 95% confidence intervals. Western blots densitometry data was analysed as a gamma (log link) glmm and results expressed as a ratio relative to β-actin using R (v 4.0.5). qPCR analysis was performed in R (v 4.0.5) using the MCMC.qpcr (v1.2.4) package^25,26^. MCMC.qpcr implements a Bayesian GLMM analysis of multi-gene qPCR data in a single model with multi-gene normalisation to endogenous controls, individual random effects for each sample, and gene-specific variances. Endogenous controls were 18S rRNA, hydroxymethylbilane synthase (HMBS) and TATA-box binding protein (TBP). Results were expressed as log2(Relative Expression).

### Ethics approval and consent to participate

Written informed consent was obtained by all participants at time of recruitment. Ethical approval was obtained from the Central Adelaide Local Health Network Human Research Ethics Committee.

## Discussion and conclusions

The airways epithelium is uniquely complex due to the range of activities required to maintain an extensive interface with the external environment and provide the conditions needed for efficient external respiration. Central to this, AEC have a range of functions that govern innate and adaptive immunity^27^. Fundamental to these phenomena is a selectively permeable barrier that protects (and is protected by) the airways via coordinated mucociliary interactions, which also surveille the atmosphere with an array of pattern recognition receptors^1^. Consequently, pulmonary function (ergo immediate life), relies on inter-AEC communication and the relay of environmental stimuli to professional immune cells that are proportional and specific to potentially harmful inhaled particles. Accordingly, the central mechanisms linked to incurable respiratory diseases such as asthma and COPD are frequently found to originate from dysfunction in the homeostatic and regenerative potential of the airway epithelium^28^. Assessing discrete disease-related phenomena in the clinic remains challenging as the airway epithelium exhibits a complex structure–function relationship and can rapidly initiate immune hyperreactions that exacerbate disease. Organotypic models derived from clinical samples can obviate many of these issues and answer fundamental questions that are not amenable to clinic research^18,23,29^. However, sampling the lower airways remains a relatively invasive procedure that requires significant resource at the operating theatre. This can greatly limit (or prohibit) the frequency and diversity of samples necessary to realise objective outcomes.

Despite the advantages of nasal cell cultures, there is insufficient agreement to support modelling the lower airway using cells from the sinonasal cavity. Here we provide evidence that organotypic systems of the nAEC and bAEC exhibit the same barrier activity within the scope of our model and analysis techniques. However, we add further evidence that there is significant uncertainty as to whether predictions can be made about gene activity in the lower airways using nAEC models.

We chose to assess barrier function as most activities governed by the airway epithelium emerge from effective cell-to-cell interactions. Also, it is reasonable to assume that the similarities between the sinonasal and bronchial epithelial architecture arise from analogous underlying regulatory mechanisms, which serve to minimise uncertainty related to these complex processes. Routine laboratory observations showed similar growth and morphological changes during progenitor cell differentiated (into a mature epithelium), which were supported by histological, immunofluorescence and high power microscopy endpoint measures (Fig. 1). Important to find a consensus between the modest number of reports in this area, our measures for growth, morphology and ciliogenesis (for the most part) align with observations made using diverse clinical and laboratory models of the airway epithelium^1,16,17,20,30^. However, endpoint analyses provide limited information about the processes that potentiate the mucociliary phenotype. In line with this, we observed higher resistance measures in nAEC during the 0–16 day differentiation interval (Fig. 2). This may reflect differences in TJ synthesis that favour nAEC, which is consistent with the heightened resilience that is ascribed to the sinonasal epithelium (e.g.^15^). Conceivably, accelerated TJ formation between nAEC would expedite healing (hence, return of function) and sentinel activity to protect the lower airways. Indeed, rapid TJ synthesis is a likely contributing factor in the maladaptive response observed in chronic rhinosinusitis that promotes nasal polyposis^31^; a phenomenon that is not observed in the lungs. While we were unable to spare cultures to assess the abundance of Claudin-1, Occludin-1 and ZO-1 corresponding to each resistance measure, we found that (in agreement with the outcomes for TEER), the abundance of these essential TJ factors were similar in both cultures (Fig. 3A). Likewise, nAEC and bAEC taken directly from biopsies (i.e. from the cytology brush in vivo) expressed approximately the same amount of TJ proteins (Fig. 3B), and the relative abundance of Claudin-1, Occludin-1 and ZO-1 were similar to those observed in the ex vivo models (Fig. 3). Hence, our results support organotypic models of the sinonasal epithelium as a reliable proxy to predict barrier function in their bAEC counterparts. However, comparisons made prior to full differentiation may be confounded by differences in the rate of TJ synthesis.

In contrast, we did observe variation in gene activity between the two epithelia for inflammatory factors commonly assessed as markers of disease at the airway epithelium (Fig. 4). The tendency for dichotomous results favouring increased transcript abundance of *TLR-4*, *IL-6*, *TNFα*, *MMP9*, and *VEGF* in nAEC generally agreed with previous reports (e.g.^16,17^). Further to these, we observed significant elevation (in nAEC cultures) of the influential regulators of airway inflammation *TGF-β* and *IL-1β* (Fig. 4). Most of these inflammatory factors participate in early responses to microbial and allergic challenges imparted by the environment^31^. So we cannot rule out that their elevation in nAEC may reflect the sinonasal labyrinth as the front-line defence against randomly encountered airborne factors (although our participants were asymptomatic), rather than any systematic difference. This is underscored by the wide confidence intervals for the majority of the inflammatory factors (with the exception of *TSLP*), and difficulties inherent to controlling environmental exposures encountered by study participants^1^. Hence, it appears important to consider which genes (or gene networks) best apply to inflammation in the lungs when using nAEC as a surrogate model. To support this recommendation, we also assessed autophagy, which is an ancient and evolutionarily conserved cellular survival process, and unlike inflammation, is a fundamental aspect of all eukaryotic cells^33,34^. In contrast to the outcomes for inflammation, there was appreciably less variation among autophagy genes (n = 29) vs the genes involved in the immunological response (n = 11). A central reason for this is autophagy is primarily an intracellular process and generally only participates in activities elicited by exogenous stimuli when they become intracellular or they restrict nutrient availability. In contrast, inflammation is orchestrated at an organismal level and requires a variety of cells (to name one intricacy). This involves complex gene-environment interactions that lend to uncertainty, particularly when trying to assign clinical or biological relevance using a data-driven approach^35,36^. Hence, validation studies are advised for particular biochemical/physiological phenomena when considering the transcriptome profile of nAEC as a means to predict gene-activity in the lungs.

Assessing the sinonasal compartment for the presence of SARS-CoV-2 has demonstrated the exceptional utility of sampling the upper airway (vs the lungs or peripheral blood), as a non-invasive means to diagnose respiratory disease. But there remains an urgent need for similar methods that predict the onset of chronic lung diseases before they become irreversible. Here, we have shown that differentiated models of the sinonasal epithelium are an effective proxy to assess barrier activity in analogous bronchial systems. While the abundance of ciliated cells are central for restricting environmental agents, we did not directly assess the important contribution imparted by the basal progenitor cells^37^, which also give rise to (for example) secretory cells that maintain the airway surface liquid^38,39^. We are now trying to determine whether barrier dysfunction in nAEC is predictive of COPD, which will incorporate a comparison of the basal and secretory cells as they are principal components for this lower airway disease. We also advise that, at present, hypothesis-driven approaches to measure gene activity are an important source of reproducible data, and that autophagy is an example where gene-activity (at least in healthy individuals) is stable at the mRNA level. While a common aetiology remains elusive, there is a clear correlation between the incidence of chronic rhinosinusitis and lung diseases with an significant genetic component such as asthma. Further, there is evidence that points towards the airway epithelium as a conduit for disease-propagation between distant sites of the respiratory tract in conditions that manifest primarily as a result of exogenous factors^5,40^. Progress in this domain can be well served in controlled laboratory systems, particularly as organotypic ex vivo models are becoming standardised and more reproducible. Outcomes from these efforts could significantly bolster renewed consideration to assess the sinonasal epithelium to expedite innovative strategies for fatal lung conditions. One ideal outcome would be the development of a cogent sinonasal transcriptome signature that enables early diagnosis and personalised treatment strategies for lung disease.

## Supplementary Information

Supplementary Information.

## Data Availability

Datasets used and analysed are available from the corresponding author upon reasonable request.

## Abbreviations

nAEC: Sinonasal airway epithelial cell
nAEC: Bronchial airway epithelial cell
AEC: Airway epithelial cell
H&E: Hematoxylin and eosin
TJ: Tight junction
TEER: Trans-epithelial electrical resistance
SEM: Scanning electron microscopy or standard error of the mean
