# Supplementary material for: Organotypic sinonasal airway culture systems are predictive of the mucociliary phenotype produced by bronchial airway epithelial cells
Source: Sci Rep. 2022 Nov 10;12:19225. doi: 10.1038/s41598-022-23667-y (PMC9648462; doi:10.1038/s41598-022-23667-y)
Supplement: Supplementary file 1 — Supplementary Information. [file 41598_2022_23667_MOESM1_ESM.pdf]

Supplementary Table. S1

Gene Name	Gene Name Full	TaqMan ID
TFEB	Transcription Factor EB	Hs00292981_m1
ZKSCAN3	Zinc Finger With KRAB And SCAN Domains 3	Hs00383244_m1
HIF1A	Hypoxia Inducible Factor 1 Subunit Alpha	Hs00153153_m1
I-CAM	Intercellular Adhesion Molecule 1	Hs00164932_m1
FOXO1	Forkhead Box O1	Hs00231106_m1
EPG5	Ectopic P-Granules Autophagy Protein 5 Homolog	Hs01125502_m1
Rubicon	Rubicon Autophagy Regulator	Hs00943570_m1
LAMP2	Lysosomal Associated Membrane Protein 2	Hs00174474_m1
LAMP3	Lysosomal Associated Membrane Protein 3	Hs01111316_m1
CALCOCO2	Calcium Binding And Coiled-Coil Domain 2	Hs00977443_m1
RNA18S5	RNA, 18S Ribosomal 5	Hs99999901_s1
NBR1	NBR1 Autophagy Cargo Receptor	Hs00245918_m1
TAX1BP1	Tax1 Binding Protein 1	Hs00195718_m1
LRSAM1	Leucine Rich Repeat And Sterile Alpha Motif Containing 1	Hs01023449_m1
MFN2	Mitofusin 2	Hs00208382_m1
ULK1	Unc-51 Like Autophagy Activating Kinase 1	Hs00177504_m1
UKL2	Unc-51 Like Autophagy Activating Kinase 2	Hs00979043_m1
ATG5	Autophagy Related 5	Hs00169468_m1
ATG7	Autophagy Related 7	Hs00893766_m1
ATG16L1	Autophagy Related 16 Like 1	Hs01003142_m1
BRD4	Bromodomain Containing 4	Hs04188087_m1
Rab1A	RAB1A, Member RAS Oncogene Family	Hs00800204_s1
Rab5A	RAB5A, Member RAS Oncogene Family	Hs00702360_s1
Rab7A	RAB7A, Member RAS Oncogene Family	Hs01115139_m1
BECN1	Beclin 1	Hs01007018_m1
ATG3	Autophagy Related 3	Hs00223937_m1
ATG12	Autophagy Related 12	Hs01047860_g1
ATG14	Autophagy Related 14	Hs00208732_m1
ATG4B	Autophagy Related 4B Cysteine Peptidase	Hs00367088_m1
PIK3C3	Phosphatidylinositol 3-Kinase Catalytic Subunit Type 3	Hs00176908_m1
GATA1	GATA Binding Protein 1	Hs01085823_m1
VMP1	Vacuole Membrane Protein 1	Hs00978589_m1
GABARAP	GABA Type A Receptor-Associated Protein	Hs00925899_g1
IFNG	Interferon Gamma	Hs00989291_m1
TNFα	Tumor Necrosis Factor	Hs00174128_m1
IL6	Interleukin 6	Hs00174131_m1
IL1B	Interleukin 1 Beta	Hs01555410_m1
CXCL8	C-X-C Motif Chemokine Ligand 8	Hs00174103_m1
TSLP	Thymic Stromal Lymphopoietin	Hs00263639_m1
MMP9	Matrix Metallopeptidase 9	Hs00957562_m1
VEGFA	Vascular Endothelial Growth Factor A	Hs00900055_m1
TLR4	Toll Like Receptor 4	Hs00152939_m1
TLR2	Toll Like Receptor 2	Hs02621280_s1
TGFB1	Transforming Growth Factor Beta 1	Hs00998133_m1
IL33	Interleukin 33	Hs04931857_m1
IL10	Interleukin 10	Hs00961622_m1
HMBS	Hydroxymethylbilane Synthase	Hs00609297_m1
TBP	TATA-Box Binding Protein	Hs00427620_m1

Supplementary Fig. S1

nAEC **bAEC**

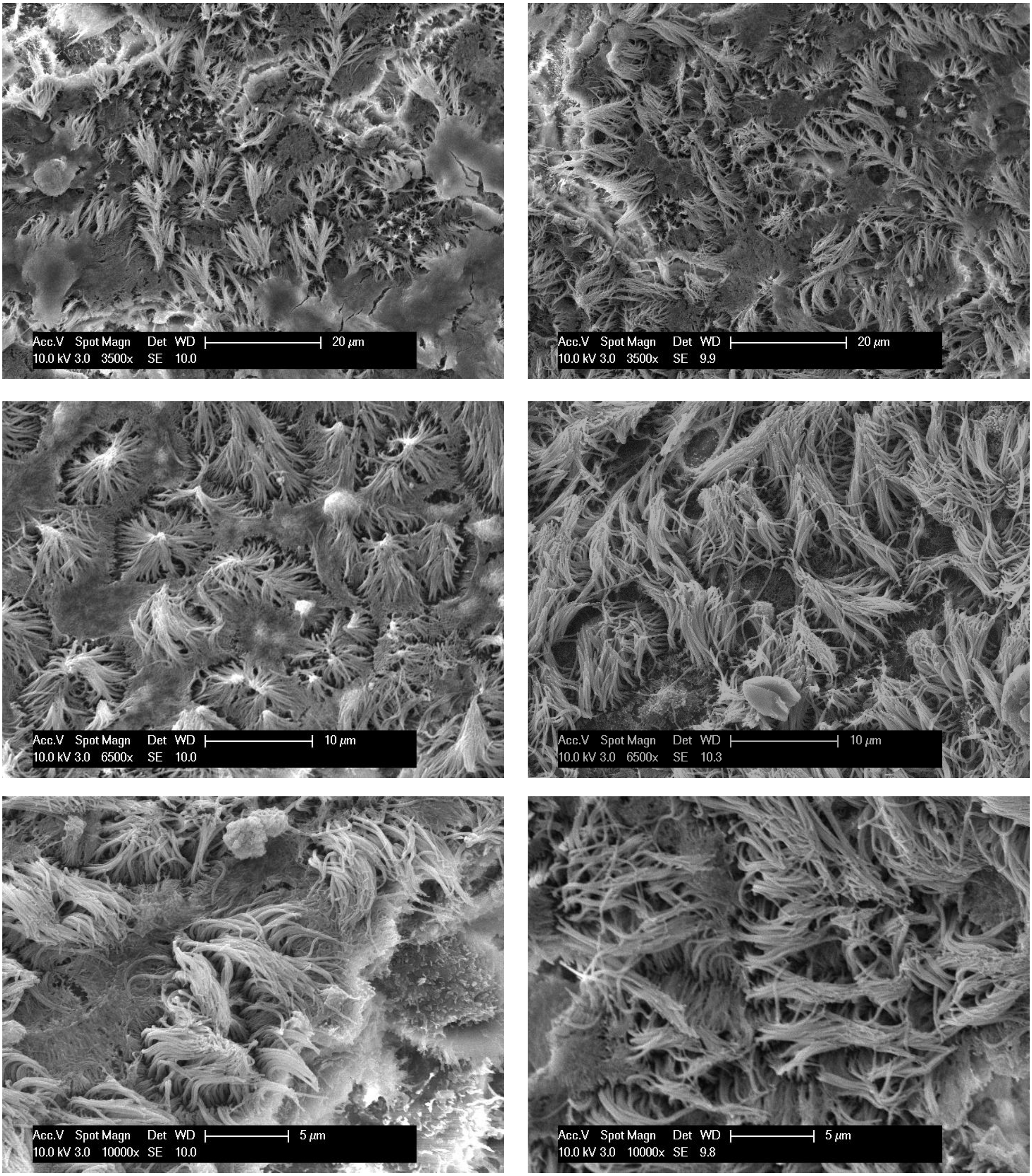

Supplementary Fig. S1: Scanning electron micrographs of nasal and bronchial cultures. Scanning electron micrographs showing two further participant nasal airway epithelial (left) and bronchial airway epithelial (right) cultures. Three increasing magnification images are shown from different fields of view.

Supplementary Fig. S2

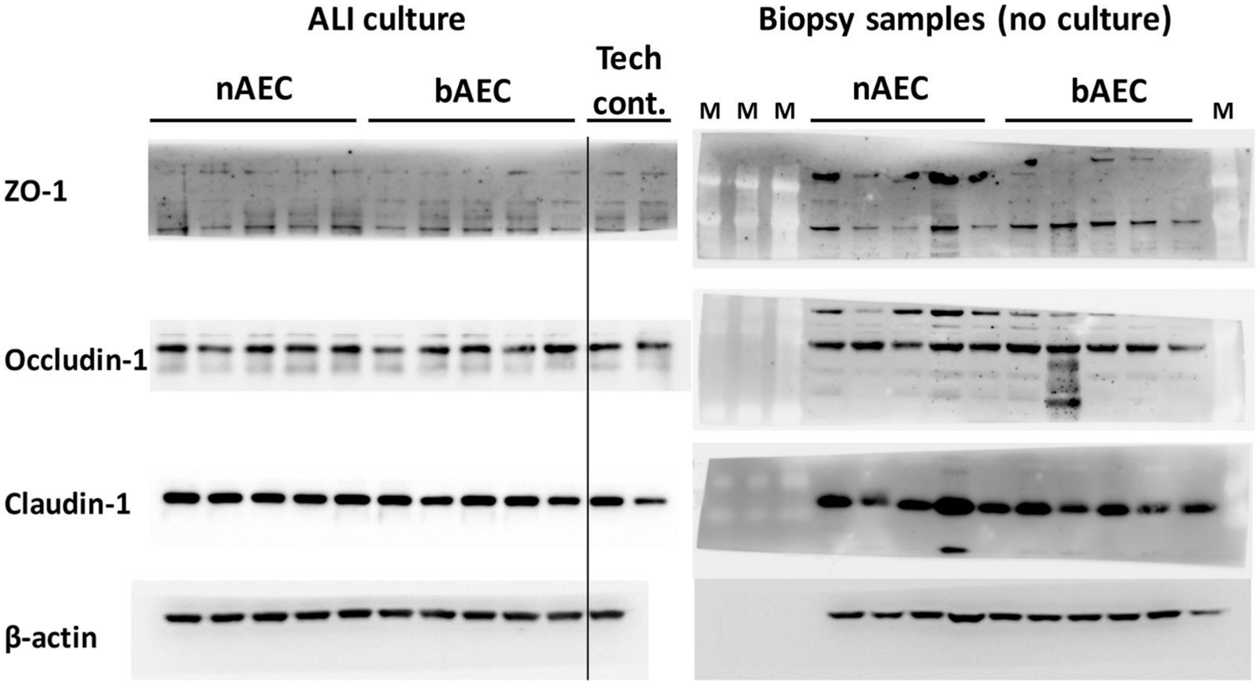

Supplementary Fig. S2: Original blots corresponding to Fig. 3. Shown are two technical controls used for outcomes related to Figure 3A for the cultured airway epithelia cells. Lane 11 (second from the right) is a positive control sample made from protein lysate from a previously generated air-liquid interface cultures derived from a commercially sourced primary airway cell (Normal Human Bronchial airway epithelial cells ("NHBE"); Lonza, Australia) that were propagated at an air-liquid interface for 32 days. This provides a standard for tight junction proteins that approximate fully differentiated primary human derived airway cells. Lane 12 (far right hand side) is protein lysate from a previously generated air-liquid interface culture also derived from NHBE cells (Lonza, Australia) that were propagated at an air-liquid interface for 2 days. This provides a standard for tight junction proteins that are partially differentiated in terms of time of epithelial cell exposure to air (vs the conventual 28+ days for complete differentiation). Marker bands are routinely removed before imaging in culture-based experiments to prevent issues related to secondary antibodies binding to the marker protein and competing with the target bands for chemiluminescence signal. The marker lanes (denoted "M") were kept/imaged for the biopsy samples due to the relative complexity of protein isolates derived from biopsy (vs cultured) samples. This allowed the target protein band molecular weight to be positively identified from spurious signals that are common in complex protein isolates from clinical biopsy specimens."
